# Registered nurses' emotional responses to medication errors and perceived need for support: A qualitative descriptive analysis

**DOI:** 10.1111/jan.16280

**Published:** 2024-06-19

**Authors:** Sanu Mahat, Anne Marie Rafferty, Katri Vehviläinen‐Julkunen, Marja Härkänen

**Affiliations:** ^1^ Department of Nursing Science University of Eastern Finland Kuopio Finland; ^2^ Florence Nightingale Faculty of Nursing, Midwifery & Palliative Care King's College London London UK; ^3^ Research Centre for Nursing Science and Social and Health Management Kuopio University Hospital, Wellbeing Services County of North Savo Kuopio Finland

**Keywords:** medication error, negative emotions, nurses, second victim, support

## Abstract

**Aims:**

To identify the contributing factors behind the second victim phenomenon, describe the emotional responses of nurses after medication errors, assess the support received by them after errors and recognize the need for a suitable support program for second victims.

**Design:**

Qualitative descriptive design.

**Methods:**

Eleven in‐depth semi‐structured interviews were conducted among registered nurses studying advanced degrees at a University in Finland during November 2021–April 2022. Data were analysed using thematic analysis.

**Results:**

The study results revealed four themes with various sub‐themes which included: contributing factors behind the second victim phenomenon; emotional responses of nurses after error; support received by nurses; and the desired need for a support program for second victims. The severity of the error and the negative work environment acted as catalysts for the second victim phenomenon among nurses. A “bitter aftermath” of emotions and a sense of insufficient support added further risk to already stressed and anxious nurses.

**Conclusions:**

This study identifies the early exploratory and enduring impact of memories associated with medication errors, some of them haunting nurses for long periods of time. Further, the need for support at different levels is highlighted to reduce the impact of negative emotions generated among nurses after medication errors.

**Implications for the Profession:**

Through the lens of this study, it has been possible to identify contributing factors behind the second‐victim phenomenon and enduring symptoms that make nurses vulnerable to becoming second victims of medication incidents.

**Impact:**

This study addresses the aftermath effect of medication errors from the perspective of nurses involved with such incidents. It provides valuable insights for healthcare managers and nurse leaders to establish a just and blame‐free culture in healthcare organizations and help emotionally traumatized nurses cope effectively after error.

**Reporting Method:**

The research adheres to Consolidated criteria for reporting qualitative research (COREQ) guidelines.

**Patient or Public Contribution:**

No patient or public contribution.

## 1 INTRODUCTION

A major turning point in the field of patient safety was observed after the publication of the Institute of Medicine (IOM), ‘To Err is Human’ which provided the initial estimates of healthcare errors associated with patient deaths. The study found preventable errors to be responsible for approximately 44,000–98,000 hospital deaths per annum in the United States (IOM, 2000).

Despite the best efforts placed by nurses and other healthcare professionals, adverse medical events sometimes occur and, in certain instances, also cause harm to the patient (IOM, 2000). Healthcare professionals might also get harmed after an adverse event, however many of them are scared or reluctant to talk about it and seek support regarding it. The possible reasons identified range from unavailability of support structures to concern about possible legal action and fear of being negatively judged by colleagues (Aljabari & Kadhim, 2021).

## 2 BACKGROUND

Patients and their families are the most immediate victims of medical errors (i.e., first victims). However, adverse events can also affect two other types of victims: the healthcare workers involved in the error and the healthcare institution where the error happens, making them the second and third victims of healthcare errors, respectively (Scott et al., 2010).

According to a survey, among 88% of surgical residents impacted by medical error, 86.5% of those residents reported experiencing subsequent emotional sequelae, such as guilt, anxiety and insomnia, or even questioning their ability to perform their work responsibilities (Khansa & Pearson, 2022). However, due to a recent debate regarding the term “second victim” (Clarkson et al., 2019; Tumelty, 2021), the use of this term is viewed by some as a way in which health professionals can evade responsibility and accountability for medical errors. Also, it might be offensive to affected patients and families (Clarkson et al., 2019) and uncomfortable for some health professionals as well to be labelled as victims (Tumelty, 2021). However, in this study, the term ‘second victim’ is used throughout the study as it connotes urgency. With increasing visibility of the importance of emotional support for health professionals, the terminology “second victim” likely will change and evolve in the future (Wu et al., 2020). Second victim syndrome (SVS) can be defined as healthcare professionals being traumatized by the error they have made manifesting psychological, cognitive and physical reactions that negatively affect their personal lives (Wu, 2000).

Medication administration (MA) is an integral part of nursing work as nurses spend a large part of their time managing medication administration (Michel et al., 2021). A high rate of stress and burnout in nurses' work environment has been identified by a National Health Services (NHS) survey conducted in 2019 in England which found 40.3% of healthcare workers experiencing burnout at their workplace (NHS, 2019). This results in an increase in the occurrence of errors and adverse events in healthcare, which in turn increases the possibility of nurses becoming mentally exhausted. Healthcare professionals who are burnt out are more likely at risk of error‐making (Royal College of Midwives, 2020). With the everyday occupational risks associated with adverse events, nurses are more likely to experience mental trauma, making them ‘second victims’ (Bruyneel et al., 2022). Kable et al. (2017) described that after being involved in adverse events, nurses experienced fear, guilt, shame, repetitive recall of the event and intensified awareness or hypervigilance, which are consistent with symptoms of post‐traumatic stress disorder (Wu et al., 2018). Similarly, not addressing this issue at the earliest opportunity can cause healthcare workers including nurses to lose their professional confidence, with some leaving their profession. Such stressors may also increase their chances of making another error, thus elevating the risk to patient safety (Burlison et al., 2021). Organizational support has been found to be of utmost importance to help healthcare workers cope effectively and be able to resume their normal duties more safely and effectively (Kable et al., 2017). A culture of openness and justice must prevail in healthcare organizations to begin an open discussion about the magnitude of the problem as well as its impact (White & Delacroix, 2020).

Unfortunately, the mental health of nurses providing care to the patient is still not viewed as a priority. Healthcare research and scientific publications addressing the concept of the ‘second victim’ and the psychological status of nurses in Finland are sparse to date. This reflects a lack of knowledge and prioritization of this issue. A gap in the literature exists regarding the actual feelings of nurses who have undergone ME incidents and regarding the support they have expected and received during that time. In Finland, this will be one of the first studies to investigate the emotional responses of nurses after making MEs. Additionally, this study explores the existing but somehow hidden second victim phenomenon among Finnish registered nurses.

## 3 THE STUDY

### 3.1 Aims

This study aimed to identify the contributing factors behind the second victim phenomenon, describe the emotional responses of nurses after medication errors, assess the support received by them after errors and recognize the need for a suitable support program for second victims. The following research questions were addressed by this study:

1. What are the main contributing factors behind the second victim phenomenon among nurses?
2. How did the errors affect nurses emotionally?
3. What kind of support did they receive after MEs?
4. How do nurses feel about the need for a support program at their institution and how it can be designed?

### 3.2 Study design

A descriptive qualitative design was used to answer the research questions. The research adhered to the Consolidated Criteria for Reporting Qualitative Research (COREQ) (Data S1) guidelines (Tong et al., 2007).

### 3.3 Sampling and recruitment

This study was conducted among registered nurses who were studying at an advanced level (either master's or doctoral degrees) at one of the Finnish universities. Recruitment was done via a convenient sampling method with the goal of acquiring more in‐depth information related to the main aims of the study (Etikan & Bala, 2017). Eligible participants were invited by posting an invitation flyer at the University's online discussion groups (Yammer).

Inclusion criteria for the participants were that they (1) were registered nurses; (2) had at least a year of working experience as a registered nurse in Finland; (3) were frequently involved in medication administration processes and (4) had experienced MEs in their working career. The participants who were willing to take part in the interview were further screened through email to confirm their eligibility.

Out of the 17 responses received, six respondents declined to participate. Among the declined nurses, four of them did not want to relive those painful moments again, whereas two reasoned as not being comfortable to share their experiences. Therefore, 11 interviews were conducted. Participants represented five different healthcare settings, two university hospitals and three different private healthcare organizations located in different parts of Finland. Informed consent and an interview guide along with an individual interview link (Microsoft teams) was then sent to participants through email. Moreover, the time and date for the interview were arranged and adjusted based on the convenience of the participants.

### 3.4 Data collection

Based on a literature review of nursing staff responses to medication error aftermath effects, a semi‐structured interview guide (Appendix A) was developed (Burlison et al., 2021; Choi et al., 2022; Ullström et al., 2014; Wu et al., 2020). The interview questions were tested by conducting a pilot interview with two participants to test the interview guide (for possible adjustments) before being tweaked for the final interview guide to ensure the richness and meaningfulness of the insights as well as ensuring whether the schedule was clear and understandable. Pilot interview data is not included in the results.

The first author carried out the interviews from November 2021–April 2022. Due to the ongoing Covid‐19 pandemic, interviews were conducted in a virtual environment using Microsoft Teams. Each interview lasted 35–60 min. Prior to the study, there was no relationship between the participants and the researcher. At the beginning of each interview, participants were informed about the study and it was ensured that they understood the purpose and procedures. Interviews were conducted in the English language which was not a native language for all the participants, however, they were fluent English speakers. The interviewer (first author) was a female doctoral researcher in nursing science and was experienced in conducting qualitative research. Before the commencement of the interview, demographic information of the participants was collected, which was followed by in‐depth semi‐structured interviews. In‐depth semi‐structured interviews were chosen as the subject area was sensitive.

Interviews were recorded using Microsoft teams and an additional audio tape for minimizing the risk of loss of data. Interview recordings were periodically reviewed by researchers (first and last author) to ascertain whether data saturation had been achieved. After eight interviews, the data became repetitive and no new theme emerged. Three more interviews were conducted with the nurses to ensure that no new information was received. As there were no new data arrived in the last three interviews, it was then ascertained that the data saturation was achieved. Therefore, no new participants were sought.

### 3.5 Data analysis

The interviews were audio‐recorded and stored by the first author, with each interview transcribed verbatim for coding and analysed by the same researcher. During transcription, the intent was to ensure that all audio recordings were transcribed in their original form to ensure a verbatim version of the discussion. There was a total of 281 pages (A4) of printed text from transcriptions.

The thematic analysis was used to analyse the data using the six‐phase framework (Braun & Clarke, 2021). In the first phase, familiarization with data was done by reading the transcripts and listening to the audio recording multiple times. In the second phase, coding was done and initial codes were produced. In the third phase, based on the produced codes, initial themes and sub‐themes were developed. Then, in the fourth phase, all the authors reviewed and refined the generated themes and related sub‐themes to ensure that they were rooted in the key purpose of the study and accurately represented the data. An iterative process of semantic analysis using an inductive approach was adopted within the data corpus until theoretical saturation was reached. All authors contributed equally to naming and describing the themes and sub‐themes in phase five and the report was produced in the sixth phase.

### 3.6 Ethical considerations

This research was carried out in accordance with the Declaration of Helsinki and it followed relevant institutional guidelines and regulations. Permission for conducting research with registered nurses enrolled in advanced degrees (Masters' and PhD) was obtained from the Department of Nursing Science, Faculty of Health Sciences, University of Eastern Finland via written statement (15 June 2021). Sufficient information about the study was given to all potential participants and both oral and written informed consent was obtained from each participant. Hence, according to the Finnish Advisory Board on Research Integrity, no ethical statement was required for this study as the research did not pose any psychological and physiological threat to the participants, followed the principle of informed consent and did not intervene in the personal integrity of the research participants (Finnish National Board on Research Integrity TENK, 2019). Further, the anonymity and confidentiality of the data and the participants were fully assured throughout the whole research process. The right of participants to refrain from answering certain questions and to end the interview process at any point if they wish was also clarified, and ethical principles of research were followed strictly (Finnish National Board on Research Integrity TENK, 2019).

### 3.7 Rigour

To ensure rigour in analysis, credibility, transferability, dependability and confirmability were established (Lincoln et al., 1985). Accuracy and consistency of the collected data were ensured by using automated Microsoft Teams generated interview transcripts and audio recordings of interviews which allowed for listening to audio tapes repeatedly throughout the study period. The credibility of the study was obtained through debriefing and review of collected data and by comparing initial findings with raw data. Further, the transferability and confirmability of the study were ensured through detailed reporting of study participants, data collection procedures and data analysis process. Similarly, regular meetings and discussions among authors helped to ensure dependability by allowing for review and reflexivity.

## 4 FINDINGS

### 4.1 Participant characteristics

The study included 11 nurses from Finland with at least one year of work experience as registered nurses in Finnish healthcare institutions. The mean age of the participants was 37.2 years and they had an average work experience ranging from 12.9 years. All the participants were females. Sample characteristics are presented in Table 1.

**TABLE 1:** Participant characteristics.

Parameter	Frequencies
Age	
25–35	6
36–45	4
46–55	1
Gender	
Male	0
Female	11
Profession	
Registered nurse studying at Masters' level	3
Registered nurse studying at Doctoral level	8
Numbers of years of experience	
1–5	4
6–10	2
11–15	1
16–20	0
21–35	3
26–30	1

### 4.2 Themes overview and description

**FIGURE 1:**
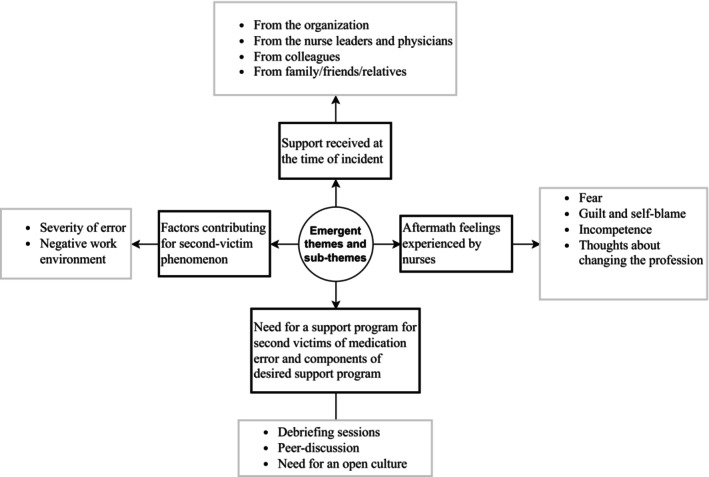
Themes and sub‐themes.

There were altogether 4 main themes generated with various sub‐themes (Figure 1).

#### 4.2.1 Theme 1: Factors contributing to the second victim phenomenon

Theme 1 consisted of two sub‐themes: (1) Severity of error and (2) Negative work environment.

##### Sub‐theme 1: Severity of error

The severity of the error was seen as a factor that impacted the strength and duration of negative emotions among the participants. Near misses and less severe errors with no significant patient harm were found to have a minimal emotional impact on nurses, helping nurses to normalize the event & hence preventing them from becoming the second victims.

Of course, it took some time for me to forget about it. It was in my mind for few days. I was thinking why I did, that kind of silly mistake. Then I rationalized it myself, gave a reason to self that there was a new staff member, she was messing with medicines here and there, so I was also under pressure which helped me to ease my feelings for a while. Well, medication error happens every now and then. (I‐8)

However, when nurses experienced severe errors, it stayed with them, impacting them for a long time. They could not stop thinking about the near‐death situation that could have happened if the patient was not monitored shortly after the medication process.

I was just thinking, what would have happened if my colleague did not check on patient for let's say till one hour and he would have already been dead… the patients' wife did ask to talk to me. I did have a discussion with her which was very hard for me. She was not aggressive but was a little bit hostile. She was continuously asking how this could happen. It was very hard to answer her questions. I just answered her I do not know how it happened, we were a bit short‐staffed and stressed. (I‐1)

##### Sub‐theme 2: Negative work environment

The work environment was mentioned by several participants as a factor contributis the error as well as how the work environment had led them to suffer in silence. For instance, one participant explained how a negative attitude from a senior might undermine the reporting of errors.

One of the ward managers was very angry with one of my colleagues, saying that she was not efficient enough. She showed a negative attitude. I think that it is a wrong way to react like that. It affects the error reporting. It just blocks the conversation regarding the error situations and nobody will have the courage to report the errors and we cannot learn anything with this. (I‐2)

Some of the nurses also stated that instead of understanding the root cause of the error, their work colleagues blamed them for not being careful about their tasks:

Physician who intubated the patient blamed us for carelessness and said that if we would have been more careful at our work, error would not have happened, but he did not realize that emergency unit was short staffed and so many cases were there at the same time…

#### 4.2.2 Theme 2: Aftermath feelings experienced by nurses

Theme 2 consisted of 4 sub‐themes: (1) Feelings of fear, (2) Feelings of guilt and self‐blame, (3) Feelings of not being competent enough and (4) Thoughts about changing profession.

##### Sub‐theme 1: Feelings of fear

Some nurses mentioned that the error generated a sense of shock and fear among themselves. Some feared the situation of the patient thinking about what if something happened because of the error. Several participants demonstrated their feeling of fear by using the terms like ‘shaking, afraid, fearful, horrified, shocked, scared, frozen’.

I was feeling horrible. It was the first error I made since I graduated. Feeling was horrible, like big rock on your chest, thought what my colleagues will think about me, stressed a lot regarding that issue, thought about what to do next… (I‐6)

I remember that I was horrified and little bit frozen, I blamed myself that how could I do this…then reflected that what might have happened to patient… (I‐8)

In contrast to this, the positive attitude of a senior was found to be a factor lessening the feeling of fear among the nurses.

I was not fearful as the manager of the ward was very supportive… I was upset, but no fear… (I‐2)

##### Sub‐theme 2: Feelings of guilt and self‐blame

Self‐blaming, self‐recrimination and feelings of guilt were found to be prominent among participants. These feelings were not so much affected by the nature or severity of the error.

I might be making excuses now but that was just because of that. May be, I don't know, I just didn't check and assumed the colleague who gave me the ampule might have checked it when he took that from cupboard. But we both failed checking. But then, I was the one who administered it, so it was my mistake. (I‐7)

Some of the nurses explained their sense of disbelief when the error happened and were not ready to accept that they could have a part in such type of MEs.

I was very disappointed to see how this could happen, usually I am very careful, always double‐checking things, but this time I was blaming myself. Also, I was ashamed when I had to tell doctor what happened… (I‐11)

##### Sub‐theme 3: Feelings of not being competent enough

Some nurses also expressed reservations about their competence triggered by errors. They explained that the error had brought a feeling of insecurity in performing their nursing duties and even made them to doubt their professional competence as a nurse.

I had really, bad feelings, I am taking that kind of errors hardly. I thought it was going to decrease my nursing competence… (I‐5)

On the other hand, nurses with several years of work experience dealt with the situation in another way. One of the participants with more than 20 years of work experience stated that errors might happen sometimes due to lack of attention. However, it has nothing to do with the competence of the nurses in relation to MA practice.

I think my medication competence is very good, that error was due to some carelessness of patient identification… I do not think it had anything to do with my competence… (I‐3)

##### Sub‐theme 4: Thoughts about changing profession

The error experience triggered thoughts among participants that perhaps nursing might not be the job for them. They said that maybe they made a wrong career choice. For example, one of the participants described on how one incident has made her co‐worker to leave the nursing profession.

But my colleague was crying, completely broken, for almost an hour…After the incident, my colleague was on sick leave for few months… changed work to elderly care and then eventually left nursing… (I‐1)

#### 4.2.3 Theme 3: Support received at the time of incident

Theme 3 consisted of 4 sub‐themes: (1) Support from the organization, (2) Support from nurse leaders and physicians, (3) Support from colleagues and (4) Support from family/relatives.

##### Sub‐theme 1: Support from the organization

Most of the participants reported that there was a lack of any kind of support system from the healthcare institution they were working in when the error happened. Also, nurses pointed that their organization did not have systematic support system and routines to handle these events, and even if it exists, it is not so visible.

It would be good to have some support system, but we did not have institutional support structure… (I‐4)

I have not seen any of these on my working places… (I‐5)

In my hospital, we did not have any systematic support system…it just depends upon ward managers personal skills and quality to handle the situation… (I‐2)

##### Sub‐theme 2: Support from nurse leaders and physicians

Seniors and physicians were found to be supportive in most cases.

To be honest, the doctor was very nice. He was very calm and said to me that the patient whom I administered those wrong medications already got those… he suggested me to follow her vitals regularly and try to let her stay awake at night as far as possible. He was not angry or rude to me. (I‐9)

However, in a few cases, ward nurses and physicians reacted in completely negative manner: blaming the nurse who made the error irrespective of the work situation.

But ward nurses have different reactions. Some have very low tolerance of errors. They like to find someone to blame…according to them it's never the situation or ward environment, rush hours. It's always nurse…sometimes I never even told the ward nurse because I knew their attitude as I witnessed it through my colleagues…instead talked with the doctor…they have always been supportive. (I‐8)

Physician who intubated the patient blamed us for carelessness… (I‐1)

##### Sub‐theme 3: Support from colleagues

Colleagues were found to be very supportive in many cases. The importance of collegial support right after the errors was highlighted by many participants. For example, for some of the participants, a positive attitude of her co‐worker helped her to cope up with the situation.

When you talk with your colleagues and they say it has happened to them as well, then it is kind of consoling and helpful. (I‐5)

They were very supportive. It was like, ok, well this has happened many times, you are not the only one. It has happened to others as well. They were teaching me and said, look always remember to check first. (I‐7)

##### Sub‐theme 4: Support from family/relatives

Familial support was deemed to be helpful for most of the participants. They explained that it lightened their heart and calmed their feelings.

Familial support is very important, no matter what happens at work, they are going to support me, I am not a bad person for them…your family will never see you differently…I am the same person…good person…good daughter and sister…it is nice to have someone to care for you… (I‐8)

I discussed the incident with my husband, he understood and told me, no worries there was no harm caused, everyone makes mistake, so it helped me a lot to forget it… (I‐6)

On the contrary, given the responsibility to maintain confidentiality in the work, some did not even discuss it with their family or friends. Also, some of them did not find it useful when discussed with their family, as the difference in profession caused their partner not to comprehend their situation appropriately.

I did not discuss it with my family…as I do not think that we should talk about our work things at home… plus my husband was not from the same background…so I did not think that he would have understood… (I‐3)

#### 4.2.4 Theme 4: Identification of the need for a support program and components of a support program based on the suggestions from nurses

Theme 4 has 3 sub‐themes: (1) Debriefing sessions immediately after the incident and counselling, (2) Peer‐discussion program and (3) Need for an open culture. When asked with the participants about their views regarding the need for a support program for the second victims of MEs, all of them agreed.

Firstly, I think that we really need that kind of program. I believe that this kind of program could make it easier to bring medication errors to light and program could also demonstrate the humanity of making mistakes. Everyone makes mistakes and I think that support is needed. (I‐6)

Nurses should not be left alone. I think in every case they should be offered support, but no idea if they will use the support or refuse to take it… (I‐7)

##### Sub‐theme 1: Debriefing sessions immediately after the incident and counselling

Some of the participants pointed out the necessity for debriefing sessions after an incident takes place. They were also fearful that not talking about the incident for a long time might lead to numbness, which could be more harmful for the patient safety. However, they have mentioned that the sessions should be conducted with someone who is not from the workplace.

If you have been working for a long time, making errors…you get used to it without any support and guidance, you will go numb (do not feel anything) and I think that it is a dangerous thing. If this happens, I do not think that you will learn from the mistakes anymore, you will be like, this happens, patient did not die… which is not appropriate. So, this kind of support program will help overcome that numbness and help you to be a good nurse…’ (I‐8)

Debriefing sessions and counselling should be offered…it should be so that we will be expressing our feelings to someone neutral… Someone who is an outsider, but not at the manager level and also maybe highlight the fact that it's going to be confidential… (I‐7)

Also, some of the participants highlighted that these debriefing and counselling sessions should be conducted right after the incident happens. For them, it is emotionally very difficult for the nurse to go through the details each time it is discussed.

…I think that it should be done immediately, but if it goes longer, it might cause that the nurse will again have to remember it over and again, repeatedly … will not get a chance to forget it… (I‐3)

##### Sub‐theme 2: Peer‐discussion program

The peer‐support program was viewed differently by the participants. Some viewed it as an extremely important way to help feel “normal” and not feel victimized by the negative thoughts of the incident for a long period of time.

You could discuss with your peers regarding the error they have made and you have made…and seek solutions like how we can do things differently so that we will not repeat the mistakes again… (I‐8)

… elements could be peer support…Maybe some kind of group discussion with the professionals who have made errors… (I‐6)

Also, knowing that other nurses had also experienced making mistakes and knowing their ways to cope with the situation have been considered helpful by some of the participants.

Peer support helps a lot …Seeing other person or knowing other people making the same mistake and how they overcome with the situation helps a lot…gives you a feeling that you are not alone in this world in this situation… (I‐4)

However, some preferred that the support should be provided anonymously in a different environment rather than the same work environment.

But in case of serious incident, I would not prefer discussion in work community, especially in bad work atmosphere because it might lead to blaming and talking behind the back… Some small group might be better with confidential agreement in your support… (I‐11)

##### Sub‐theme 3: Need for an open culture

An open, just and blame‐free culture was highlighted by all participants, so that there would not be much harm caused to the second victims of MEs.

Open culture where one can share things with others…so it might ease their feelings… Many nurses do not report the errors which might cause danger to the patient…they should not think that they are a bad nurse, as it might affect the mental health of the nurse… (I‐3)

Some also suggested that the organizational and work environmental factors should be taken care of beforehand so that the errors will be minimized rather than blaming nurses for their carelessness.

Medication error is not just about nurse and medicine. It is also about working environment and work colleagues. Very minimal nurse patient ratio is affecting a lot. One nurse must take care of 10 patients and it is highly likely that error happens in these stressful hours… (I‐9)

## 5 DISCUSSION

This study aimed to identify contributing factors to the second victim phenomenon and emotional responses of Finnish nurses after making MEs, as well as the potential need for support programs and design features of such program. A notable aspect of this study is that it also validates the need for a support program for those nurses who experience MEs. This study also provides baseline data on the factors that need to be considered while developing and implementing support programs.

Along with negative emotions experienced, participants from this study also mentioned a few contributing factors that lead to those negative emotional experiences. One of these was the type and severity of errors they had made. Participants mentioned that errors of a more severe nature where the patient was in grave danger had stayed with them for a longer period. Previous research has confirmed this finding that the emotional impact of an error lasts from several weeks to several years depending upon the nature of the error (Baas et al., 2018; Choi et al., 2022). This could contribute to invasive cognitive rumination where the individuals repeatedly and consciously think about the traumatic events leading to an increase in anxiety, helplessness and trigger traumatic mood, thus negatively impacting their coping mechanisms (Wu et al., 2018). In contrast, this study found near misses and less serious errors caused less emotional harm to nurses, which has also been confirmed by a recent survey study (Choi et al., 2022). A negative work environment was mentioned as another contributing factor behind having more intense negative emotions and aftermath effects. Inadequate support and negative attitudes from seniors and colleagues seemed to deepen and prolong the impact of errors on nurses. Previous research has also shown that the impact of error depends upon the way the incident is handled. Scant support and lack of a clear investigation were found to deepen and prolong the impact (Choi et al., 2022; Ullström et al., 2014).

The second major finding was the emotional distress experienced by most of the participants following MEs. Intense feelings of fear, guilt, self‐blame and professional incompetence were expressed as experienced by nurses. These findings were similar to the findings of a study by Mahat et al. which found the feelings of fear, emotional disturbance, sadness and guilt among healthcare professionals after making MEs while analysing the medication administration error incident report from England and Wales (Mahat et al., 2022). Studies have demonstrated a greater degree of physical and psychological symptoms including consideration of job changes among those healthcare workers involved directly in patient safety incidents (Choi et al., 2020). Previous studies of second victims depict healthcare workers not to be affected only physically and psychologically but also professionally (Burlison et al., 2021; Lee et al., 2019). A cross‐sectional survey conducted in Spain discovered 6 out of 10 healthcare professionals in Spain experiencing emotional and professional difficulties either themselves or in their colleagues in the aftermath of adverse patient event. Negative emotions such as feelings of guilt, anxiety, re‐living the event, tiredness, insomnia and persistent feelings of insecurity were reported, which are all the symptoms of PTSD (Mira et al., 2015).

In contrast, participants explained that a positive attitude from their seniors and colleagues had minimized their fear. Appropriate support from others has been found to alleviate the suffering of healthcare workers, while lack of support has been found to increase their psychological burden (Lee et al., 2019; Scott et al., 2010).

Although the emotional response to MEs was more severe at the time of the incident, it was found that these incidents continued to affect nurses even in the long run. Research into second victim recovery trajectory by Lee et al. (2019) have reported that despite healthcare professionals' consistent efforts to overcome the impact of errors, emotional wounds inflicted on them due to errors were not erased. Some nurses even choose to give up their nursing careers (Huang et al., 2020).

Emotional support has been considered important by participants stating that it helped them to cope better. A previous study implementing a peer support program for second victims of medical error also stressed the importance of supportive intervention for healthcare professionals (Edrees et al., 2016). However, most participants reported a lack of active support structure in the Finnish healthcare system whom they could turn to at the time of the incident. Nevertheless, sharing their experiences about the errors with their colleagues having experienced similar situations has helped them to improve their emotional stability which confirms previous research (Edrees et al., 2016).

Regarding the support received, most participants revealed a lack of structured and systemic support from the healthcare organization they were working for at the time of ME. Organizational support was found to be missing in most healthcare organizations worldwide as reported by a recent systematic review (Busch et al., 2021).

Support received was mainly from colleagues who had undergone similar experiences in the past and from seniors who instead of blaming the nurses, helped them to cope with the error. A shift in safety culture, in which a no blame attitude is adopted is important alongside supportive care and interventions (Ritterman, 2017). A blame culture rather than one of support can have devastating effects on the psychological state of a healthcare professional after error. This can even result in moral injury. Moral injury is described as negative mental health consequences of events that gives a feeling of violation of moral code to the healthcare provider (Kopacz et al., 2019). Second victim phenomenon can occur without moral injury (Chandrabhatla et al., 2022). However, in extreme cases, where blame culture is prevalent, moral injury is not an exception. By cultivating a just and open culture, one can minimize emotional distress related to adverse event (Van Gerven et al., 2016). However, some of the participants in this study also felt that open disclosure about the MEs was not routine in the organization they were working for.

Despite lack of complete understanding of the situation, support from family and friends was found to be important and helpful for nurses in this study: listening was especially helpful. However, some of the participants did not discuss the situation with their families because of confidentiality issues. A strong need has been identified by other research studies, where nurses can discuss the events with others, so that they can learn from it. Otherwise, they may feel isolated with their feelings and often suffer in silence (Ullström et al., 2014).

Many healthcare workers have opted for reduced shifts, transferring to other units, or changing occupations as a way of coping (Burlison et al., 2021; Lee et al., 2019) while those sustaining severe trauma quit their jobs and even commit suicide to avoid the stigma and burden of a tainted reputation, sentence, or lawsuit (Ullström et al., 2014). This further escalate the continuous and impending loss of global health workforce which is already estimated in millions. Shortage of 15.4 million healthcare professionals in 2020 was estimated globally which is one of the major challenges faced by nursing workforce as well (Boniol et al., 2022). International Council of Nurses (ICNs) in its recent report voiced that the worldwide shortage of nurses should be treated as global health emergency (International Council of Nurses, 2023).

The final findings of this study were the need for support and how the support structure in healthcare institutions for the nurses and other healthcare workers after MEs could be designed. Debriefing sessions and counselling were one of the support structures hoped for by participants. Some of the participants suggested that debriefing sessions should happen right after MEs, not after a month or year of the incident, because for some, recalling the same event repeatedly for a long period of time is traumatic. Debriefing sessions has been found to be effective in terms of immediate after‐math of MEs working as an emotional first‐aid to reduce negative feelings among ‘second victims’ (Harrison & Wu, 2017). Increased intention to leave job and increased absenteeism among nurses suffering from SVS were found to be decreased when organizations supported them through the desired response (Burlison et al., 2021).

Similarly, peer discussion with those who have had similar experience in the past and peer support programs by experienced and trained professionals was put forward by nurses in this study. They believe that this kind of peer discussion program will help them to learn coping strategies. Additionally, they will not feel left out or alone and think themselves as sole person making error. Knowing that others have also went through similar experiences helps them to cope effectively. Likely, early adopter institutions have also suggested three‐tiered support system for healthcare professionals suffering from SVS, i.e., emotional first aid by trusted colleague or mentor, support from trained peers and support by mental health professionals (Edrees et al., 2016). Sharing experiences with a non‐judgemental colleague have helped those who err to ease emotional hindrance via trust, reassurance and support (Rinaldi et al., 2022; Ullström et al., 2014).

In addition, participants in this study also desired for an open and just culture in the organization, where focus of errors will be shifted from an individual to more holistic and system‐wide perspective which might have contributed towards errors. Similar evidence could be found from other previous studies suggesting an importance of paradigm shifts from a culture of blame to fairness and need for a balanced and responsible approach to improve healthcare conditions at workplace (Petschonek et al., 2013). Adoption of a just and blame‐free culture also facilitates for disclosure of MEs which will positively impact patient safety.

To summarize, in‐depth experiences of our participants suggest that the second‐victim phenomenon is neglected in some healthcare institutions to some extent and there are only a few unstructured support programs for nurses experiencing negative emotions after getting involved in medication incidents. Our participants recommend a structured support system to be put in place in their workplace. However, given the scale and severity of this issue, more research is needed to investigate the extent of this problem and develop effective and sustainable support strategies at national level.

### 5.1 Strengths and limitations of the work

After interviewing eight nurses, data saturation was reached and only three more participants were interviewed. However, the respondents to the invitation were very few despite sending multiple reminders to increase the number of participants. However, an in‐depth understanding of the phenomenon provided by this study demonstrates the need for support, thus cannot be neglected. Our interviewees also varied in terms of age, years of experience and in the nature and outcome of MEs they have encountered with. Another limitation associated with this study is that the participants volunteered themselves for participation, so there could be an issue of self‐selection bias. Even though the participants were native Finnish speakers, the interview was conducted in English. Hence, some unseen difficulties might have been there for the participants to express their feelings in foreign language although they were fluent English speakers. Therefore, a chance of loss of complete information cannot be put aside. As the interview was conducted online using Microsoft teams due to the ongoing COVID‐19 pandemic situation, it was not possible for the interviewer to observe the non‐verbal expressions of participants as the video camera was chosen to be turned off by most of the participants. It could be understood as participants' concern about their psychological safety as some kind of fear still could have stayed with them. Further, COVID‐19 posed limitations on recruiting frontline nurses working in hospital and only graduate and post‐graduate students were recruited. This could account for the possibility of researcher bias. However, as participants represented five different workplaces, this study provide in‐depth understanding of the phenomenon in different healthcare organization within Finland. The data collected for this study also includes the description of MEs made that were made many years previously (some 15 years), when the systems of e‐prescribing and some of the modern medication practices were not available. Therefore, the current extent of problem cannot be identified from some interviews.

### 5.2 Implications for practice

The findings from this study have enhanced our knowledge of the emotional journey of nurses living with stark and intense memories of the MEs they have committed and circumstances surrounding it. It further helped us to identify the extent of this problem among nurses working in Finland, as this is one of the first study known to analyse the emotional experiences of nurses in Finland. However, given that some of the participants in this study have already stopped working in clinical setting long before, this makes it difficult to understand the magnitude of the current problem. Therefore, it is paramount to identify the problem with clinically active nurses. Through the lens of this study, it has been possible to identify contributing factors behind second‐victim phenomenon and the enduring symptoms and feelings that make nurses vulnerable to becoming second victim of medication incidents. Understanding the sensitivity of the issues and the actual emotional responses of nurses can help address similar problems in the future. Further, support programs for nurses are of paramount importance in clinical environment to help nurses as well as other healthcare professionals who are at risk of becoming second victim of MEs.

### 5.3 Recommendations for future research

Findings from this study address the lack of support structures for nurses after making medication errors, thus highlighting the need of future research addressing the necessity of structured support strategies in healthcare organizations. Rational ideas expressed by nurses regarding their desired form of support could be helpful to design an effective support program. To explore the practical requirements for designing such system, attention should be paid on work environment where these issues could be discussed without fear of being judged. Similarly, studies focusing on measures to change the negative attitude and approach of healthcare leaders and organization towards ‘second victims’ in clinical settings are needed. In addition, healthcare organizations need to understand importance of constructive communication on emotional well‐being of healthcare professionals after MEs.

## 6 CONCLUSION

Nurses in general are carrying the burden of risk of MEs as they are the main actors doing MA. As reported in this study, nurses were in some cases deeply affected by their involvement in MEs, which triggered their need for support, expectations and how well those needs were met. However, in case of errors, the system is not protective enough as it lacks safe and nonpunitive environment for error reporting. Therefore, nurses are suffering moral distress and injury despite having no fault of their own. Shifting the focus from blaming the error‐maker to finding the root cause of error and supporting nurses who err might help in building a protective system. Our findings also underline the need for the use of transparency in investigating and analysing MEs in addition with adequate continuous support from healthcare managers and colleagues for those nurses involved in MEs.

## AUTHOR CONTRIBUTIONS

All the authors made significant contributions in conception and design of the study, acquisition of data, data analysis and interpretation of findings. Drafting of the manuscript was done by the corresponding author and all the authors revised the manuscript critically and gave their final approval for the version of the manuscript to be submitted.

S.M., A‐M. R., K. V‐J., M.H.: Made substantial contributions to conception and design, or acquisition of data, or analysis and interpretation of data; S.M., A‐M. R., K. V‐J., M.H.: Involved in drafting the manuscript or revising it critically for important intellectual content; S.M., A‐M. R., K. V‐J., M.H.: Given final approval of the version to be published. Each author should have participated sufficiently in the work to take public responsibility for appropriate portions of the content; S.M., A‐M. R., K. V‐J., M.H.: Agreed to be accountable for all aspects of the work in ensuring that questions related to the accuracy or integrity of any part of the work are appropriately investigated and resolved.

## FUNDING INFORMATION

This research received no specific grant from any funding agency in the public, commercial, or not‐for‐profit sectors.

## CONFLICT OF INTEREST STATEMENT

No conflict of interest has been declared by the authors.

## PEER REVIEW

The peer review history for this article is available at https://www.webofscience.com/api/gateway/wos/peer‐review/10.1111/jan.16280.

## Supporting information


Data S1.

## Data Availability

The data that support the findings of this study are available from the corresponding author upon reasonable request.

## APPENDIX A

### A.1

Interview guide.

**Table:**

(A) Background information
How old are you? What is your academic qualification? For how long you have been working or worked as a Finnish registered nurse?
(B) Experiences of Medication Errors (MEs)/incidents
Have you ever experienced/witnessed Medication Errors (MEs)/near misses in your working life as a nurse? If yes, could you please describe the nature and severity of the error/near miss? Please describe your experiences and immediate reaction to and feelings after the incident. Did you have any fears after the incident? Please elaborate on it.
(C) Influence of MEs on your personal and professional life
5 How often do you think about past incidents at present? What kind of situations trigger your thoughts? 6 As far as you are aware, have you had any kinds of psychological symptoms? If yes, what are those? 7 How do you feel your working life as a nurse has been after the incident? 8 Has it ever disturbed/disrupted or intruded into your personal/family life?
(D) Coping mechanisms
Did you cope after the incidents? What were your coping mechanisms? If you have witnessed someone in your close acquaintance experiencing such incidents, what have you observed from them regarding their way of coping?
(E) Support received
*Employer* Was there any kind of support system/program available in your workplace to help healthcare workers aftermath ME incidents? If yes, what was it? How did it help you to overcome your negative emotions? Was the support enough for you? If not, please describe what would have been better. *Colleagues* Did you receive any kind of assurance from your co‐workers? If yes, what were they? In situations like this, what are your expectations from your colleagues? Please elaborate on your views regarding the importance of collegial support. *Family/friends/relatives* If there was any impact of the incident on your personal life, please describe it. Did you receive any support from your family/relatives? If yes, please elaborate on the kind of support you received. Describe your views regarding the role of family/relatives in restoring the personal image of those affected aftermath ME incidents.
(F) Suggestions based on your experiences
What is your view regarding the need for support programs after ME incidents? What might the elements of such a support program be? Would you be interested in being involved in the design of such a program?
