# Supplementary material for: Registered nurses' emotional responses to medication errors and perceived need for support: A qualitative descriptive analysis
Source: J Adv Nurs. 2024 Jun 19;81(9):5458–71. doi: 10.1111/jan.16280 (PMC12371863; doi:10.1111/jan.16280)
Supplement: Supplementary file 1 — Data S1. [file JAN-81-5458-s001.docx]

Supplementary file 1. COREQ (COnsolidated criteria for REporting Qualitative studies) Checklist.

| **Item No** | **Item** | **Guide questions/description** | **Reported on Page No.** |
| --- | --- | --- | --- |
| **Domain 1:**  **Research team**  **and reﬂexivity** | | | |
| *Personal*  *Characteristics* | | | |
| 1 | Interviewer/facilitator | Which author/s conducted the interview or focus group? | Methods (p.8) |
| 2 | Credentials | What were the researcher’s credentials? *E.g., PhD, MD* | Title Page |
| 3 | Occupation | What was their occupation at the time of the study? | Title Page |
| 4 | Gender | Was the researcher male or female? | Methods (p.8) |
| 5 | Experience and training | What experience or training did the researcher have? | Methods (p.8) |
| *Relationship with*  *participants* | | | |
| 6 | Relationship established | Was a relationship established prior to study commencement? | Methods (p.8) |
| 7 | Participant knowledge of the interviewer | What did the participants know about the researcher? *e.g., personal goals, reasons for doing the research* | Methods (p.8) |
| 8 | Interviewer characteristics | What characteristics were reported about the inter viewer/facilitator? *e.g., Bias, assumptions, reasons, and interests in the research topic* | N/A |
| **Domain 2:**  **Study design** | | | |
| *Theoretical*  *framework* | | | |
| 9 | Methodological orientation and Theory | What methodological orientation was stated to underpin the study? *e.g., grounded theory, discourse analysis, ethnography, phenomenology, content analysis* | Methods (p.8) |
| *Participant*  *selection* | | | |
| 10 | Sampling | How were participants selected? *e.g., purposive, convenience, consecutive, snowball* | Methods (p.4-5) |
| 11 | Method of approach | How were participants approached? *e.g., face-to-face, telephone, mail, email* | Methods (p.6-7) |
| 12 | Sample size | How many participants were in the study? | Results (p.7) |
| 13 | Non-participation | How many people refused to participate or dropped out? Reasons? | Methods (p.7) |
| *Setting* | | | |
| 14 | Setting of data collection | Where was the data collected? *e.g., home, clinic, workplace* | Methods (p.7) |
| 15 | Presence of non-participants | Was anyone else present besides the participants and researchers? | N/A |
| 16 | Description of sample | What are the important characteristics of the sample? *e.g., demographic data, date* | Results (p.7) |
| *Data collection* | | | |
| 17 | Interview guide | Were questions, prompts, guides provided by the authors? Was it pilot tested? | Methods (p.10) |
| 18 | Repeat interviews | Were repeat interviews carried out? If yes, how many? | N/A |
| 19 | Audio/visual recording | Did the research use audio or visual recording to collect the data? | Methods (p.8) |
| 20 | Field notes | Were ﬁeld notes made during and/or after the interview or focus group? | N/A |
| 21 | Duration | What was the duration of the interviews or focus group? | Methods (p.7) |
| 22 | Data saturation | Was data saturation discussed? | Methods (p.8) |
| 23 | Transcripts returned | Were transcripts returned to participants for comment and/or correction? | N/A |
| **Domain 3:**  **analysis and ﬁndings** | | | |
| *Data analysis* | | | |
| 24 | Number of data coders | How many data coders coded the data? | Methods (p.8) |
| 25 | Description of the coding tree | Did authors provide a description of the coding tree? | N/A |
| 26 | Derivation of themes | Were themes identiﬁed in advance or derived from the data? | Methods (p.8-9) |
| 27 | Software | What software, if applicable, was used to manage the data? | N/A |
| 28 | Participant checking | Did participants provide feedback on the ﬁndings? | N/A |
| *Reporting* | | | |
| 29 | Quotations presented | Were participant quotations presented to illustrate the themes/ﬁndings? Was each quotation identiﬁed? *e.g., participant number* | Results (p.10-19) |
| 30 | Data and ﬁndings consistent | Was there consistency between the data presented and the ﬁndings? | Results (p.10-19) |
| 31 | Clarity of major themes | Were major themes clearly presented in the ﬁndings? | Results (p.10-19) |
| 32 | Clarity of minor themes | Is there a description of diverse cases or discussion of minor themes? | Discussion (p19-25) |

Developed from: Tong A, Sainsbury P, Craig J. Consolidated criteria for reporting qualitative research (COREQ): a 32-item checklist for interviews and focus groups. International journal for quality in health care. 2007 Dec 1;19(6):349-57.
